# Lithotripsy-assisted TAVR for severe calcific aortic stenosis. Case report

**DOI:** 10.1093/ehjcr/ytag042

**Published:** 2026-01-27

**Authors:** Jhonathan Uribe-Gonzalez, Daniela L Leocachin-Parra, Juan F Arreguin-Porras, Miguel A Chagoya-Triana, Joel Estrada-Gallegos

**Affiliations:** Department of Interventional Cardiology, ‘Hospital de Cardiologia de Centro Médico Nacional Siglo XXI’, Faculty of Medicine, National Autonomous University of Mexico, 330 Av. Cuauhtémoc, Mexico City 06720, México; Department of Interventional Cardiology, ‘American British Cowdray Hospital’, 116 Sur 136, Mexico City 01120, Mexico; Department of Interventional Cardiology, ‘Hospital de Cardiologia de Centro Médico Nacional Siglo XXI’, Faculty of Medicine, National Autonomous University of Mexico, 330 Av. Cuauhtémoc, Mexico City 06720, México; Department of Interventional Cardiology, ‘Hospital General del Centro Médico Nacional La Raza, Faculty of Medicine, National Autonomous University of Mexico, S/N Paseo de las Jacarandas, Mexico City 02990, México; Department of Interventional Cardiology, ‘Hospital General del Centro Médico Nacional La Raza, Faculty of Medicine, National Autonomous University of Mexico, S/N Paseo de las Jacarandas, Mexico City 02990, México; Department of Interventional Cardiology, ‘Hospital de Cardiologia de Centro Médico Nacional Siglo XXI’, Faculty of Medicine, National Autonomous University of Mexico, 330 Av. Cuauhtémoc, Mexico City 06720, México

**Keywords:** TAVR, Lithotripsy, Severe calcium

## Abstract

**Background:**

TAVR has become the first option in several scenarios for the treatment of aortic stenosis. Nevertheless, calcium can lead to suboptimal outcomes concerning paravalvular leak and annular rupture. In this context, novel therapies have been emerging; intravascular lithotripsy (IVL) with Shockwave balloons is one of the most recent, with promising results, although more research is needed to propose this kind of therapy in this context.

**Case summary:**

We present the case of a 78-year-old woman with severe calcific aortic stenosis and a very small annulus, treated with intravascular lithotripsy. Sequential IVL balloon inflations (two 7.0 × 40 mm balloons, then an 8.0 × 40 mm balloon) were performed, followed by an 18 mm valvuloplasty balloon and deployment of a 23 mm Accurat Neo2. The procedure achieved an excellent final result with no complications, no paravalvular leak, and favourable early outcomes at 6 months.

**Discussion:**

This case highlights the novel use of these types of calcium-modifying devices for other than coronary and peripheral vessels with promising outcomes.

Learning points

- Transcatheter aortic valve lithotripsy can be a feasible and efficient therapy in heavily calcified aortic stenosis
- Cerebral protection devices can be considered in certain scenarios of increased neurological risk.

## Introduction

In numerous cases, *trans*-catheter aortic valve replacement (TAVR) has become the first-line option for the treatment of aortic stenosis. Nevertheless, some challenges are still encountered in the context of heavily calcified aortic valves. The major technical limitations associated with severe calcification include para-valvular leak, *trans*-catheter heart valve subexpansion or migration and even annular rupture. In recent years, the use of intravascular lithotripsy (IVL) in the aortic anulus prior to TAVR has emerged with favourable outcomes.^1–3^

## Case presentation

**Figure 1:**
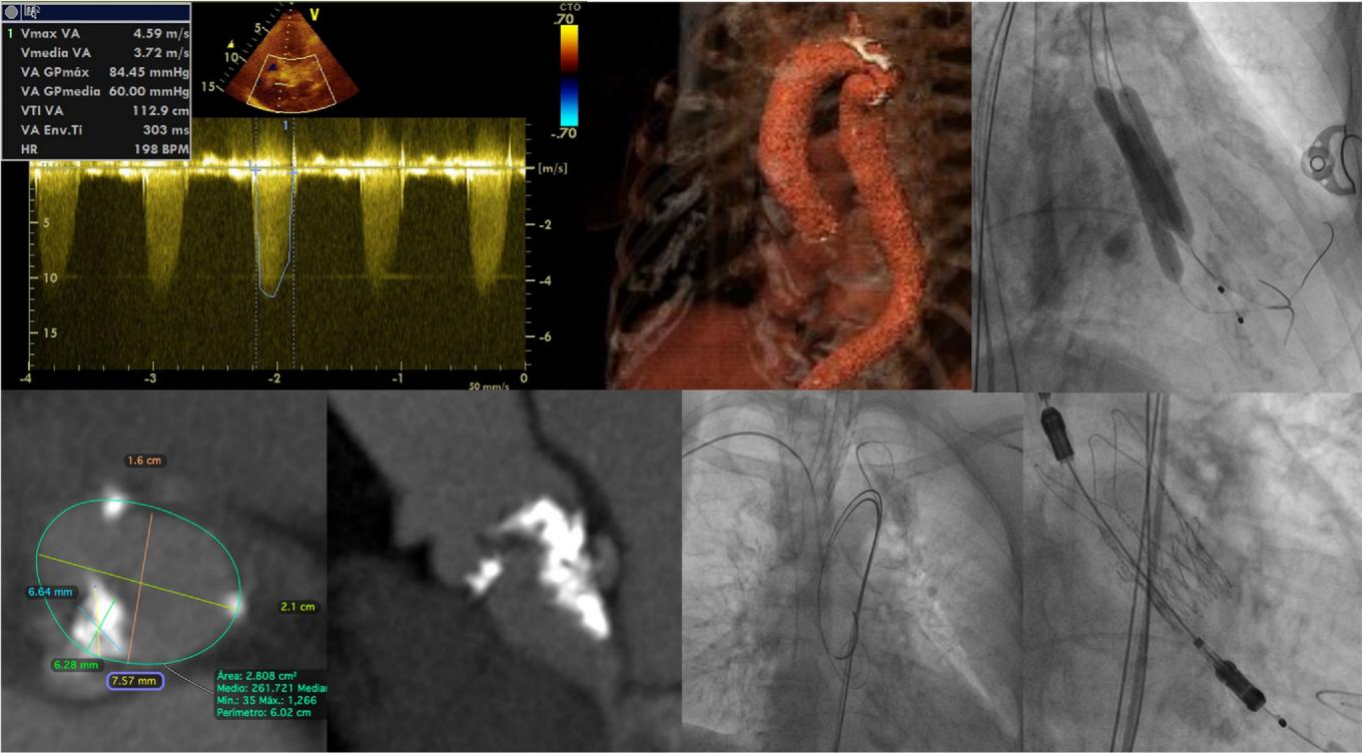
Schematic summary.

**Figure 2:**
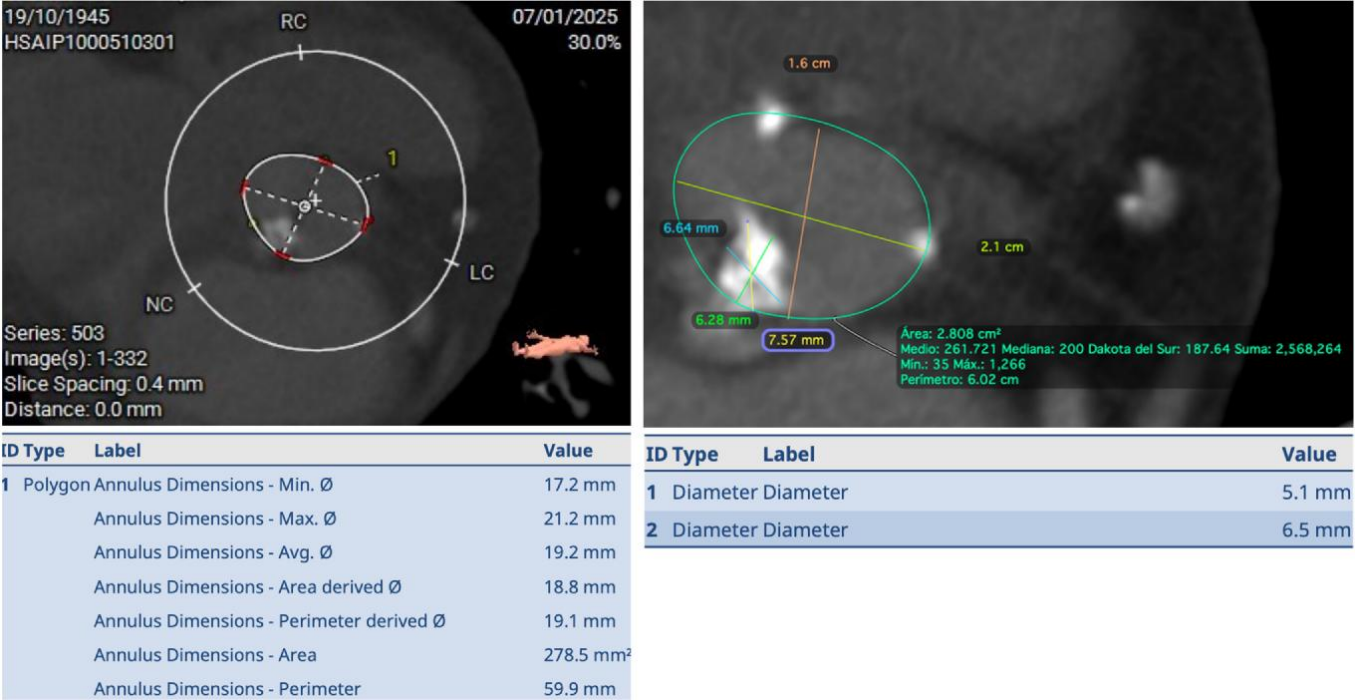
Preprocedural CT scan showing the annular measurement and calcium nodule.

**Figure 3:**
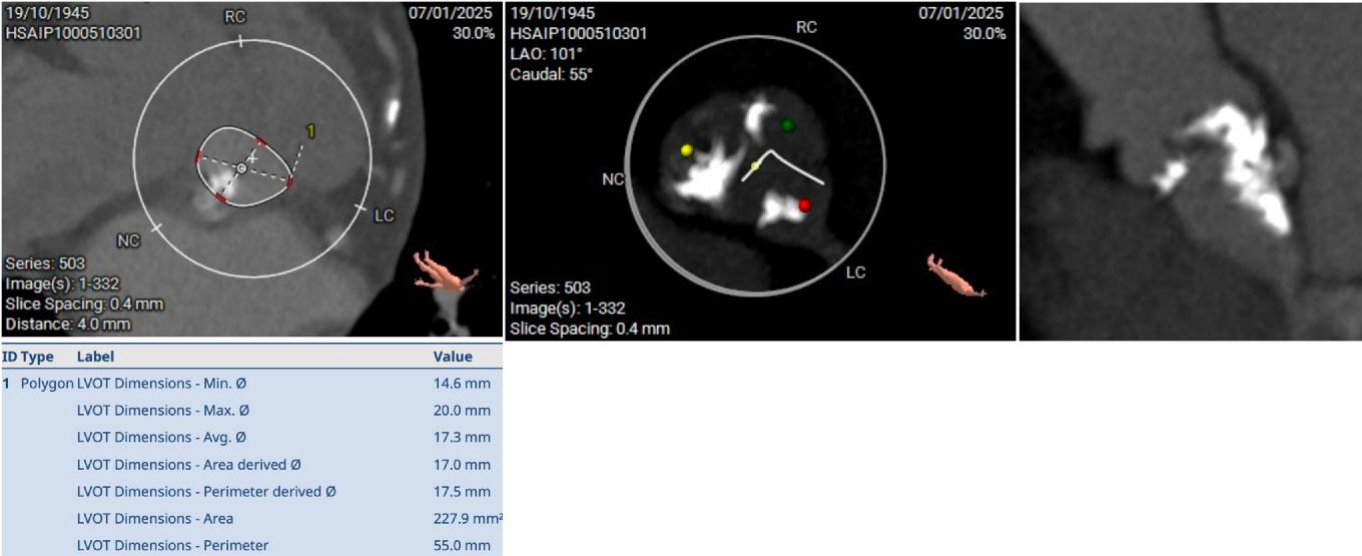
Preprocedural CT scan revealing the LVOT measurement and calcium burden.

**Figure 4:**
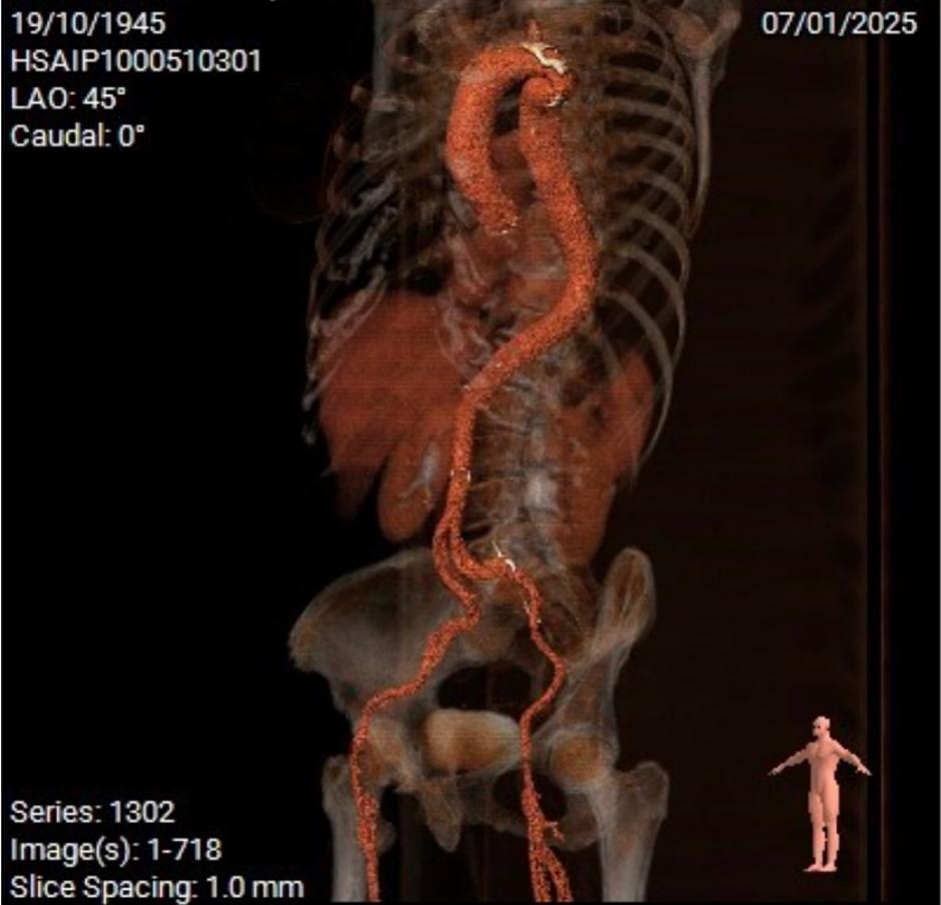
CT scan reconstruction of the aorta indicating pseudocoarctation.

A 78-year-old female patient with a history of dyspnoea and fatigue was referred to our institution. Her previous medical history included smoking and chronic obstructive pulmonary disease (COPD). A systolic aortic murmur was identified on physical examination. Electrocardiogram showed a sinus rhythm without atrioventricular or bundle branch blocks. Transthoracic echocardiogram (TTE) revealed concentric left ventricular hypertrophy with preserved left ventricular ejection fraction and severe aortic stenosis, with an area of 0.57 cm^2^, mean gradient of 60 mmHg, and maximum velocity of 4.59 m/s (*Video 1*) (*Figure 1*). Coronary angiography ruled out coronary artery disease (*Video 2*). An extremely small aortic annulus (area: 278.8 mm^2^) with a large eccentric calcium nodule (6.6 mm × 6.2 mm) was found on computed tomography (CT) scan (*Figure 2*). The left ventricle outflow tract (LVOT) and sinus of Valsalva exhibited moderate to severe calcification (2050AU), primarily located in the noncoronary sinus (*Figure 3*). Appropriate coronary ostium heights and femoral access were confirmed. Further, an aortic pseudo-coarctation was found in the descending aorta beyond the left subclavian artery (*Figure 4*). Although the patient was at low-intermediate risk (European System for Cardiac Operative Risk Evaluation II: 2.85%, Society of Thoracic Surgeons risk score: 5.29%), the presence of COPD with a severe restrictive pattern prompted the cardiology team to perform TAVR, despite the patient presenting with challenging anatomical characteristics.

The procedure was performed via the *trans*-femoral access. After crossing the aortic pseudo-coarctation with a JR-5-Fr diagnostic catheter, the triple buddy wire technique was used, positioning a Lunderquist wire and a 0.035-in Safari extra-small wire into the ascending aorta, and a 0.035-in Amplatz Superstiff wire at the left subclavian artery for further support. A Sentinel cerebral embolic protection device (Boston Scientific, Marlborough, MA, USA) was placed via the right radial access. After crossing the aortic valve, the peak-to-peak gradient was 70 mmHg. Two 300-cm 0.014-in Mailman wires were inserted into the left ventricle. Then, two 7.0 mm × 40 mm lithotripsy balloons (Shockwave Medical, Santa Clara, CA, USA) were inserted over each wire across the aortic valve using the iSLEEVE sheath (Boston Scientific, Marlborough, MA, USA). Simultaneous inflation of the two balloons was performed at 4 atm, delivering two cycles with 10 pulses under rapid pacing. Subsequently, an additional 8.0 mm × 40-mm lithotripsy balloon (Shockwave Medical, Santa Clara, CA, USA) was advanced using an 8-Fr left femoral sheath, and three cycles with 10 pulses were delivered. The subsequent peak-to-peak gradient was 30 mmHg. Therefore, the procedure was completed by performing a valvuloplasty with an 18 mm × 40-mm Valver balloon (Balton, Warsaw, Poland). Finally, an Accurate Neo2 Small 23-mm valve (Boston Scientific, Marlborough, MA, USA) was deployed (*Video 3*). The peak-to-peak final gradient was 0 mmHg. The final aortic root angiography showed no para-valvular leak without any complications.

The patient was discharged home 3 days after the procedure. At the 30-day follow-up, the patient remained asymptomatic, with the New York Heart Association functional class I status and no conduction disturbances. Based on the control echocardiogram, the mean gradient of the prosthesis was 2.2 mmHg, and no para-valvular leak was observed (*Video 4*).

## Discussion

Aortic stenosis is the most common primary valve lesion that requires intervention for definitive treatment.^4^ The current treatment guidelines recommend surgical replacement for younger patients who are at low risk and those who are operable and unsuitable for *trans*-femoral TAVR.^4^ Meanwhile, TAVR is recommended for high-risk older patients or those who are not suitable for surgery. However, recent trials have confirmed the feasibility of TAVR across the whole spectrum of surgical risk.^5,6^ In this context, challenging cases are becoming increasingly common.

Severe calcified aortic stenosis may lead to several technical limitations and poor outcomes. To manage these cases, novel therapies have been emerging. Intravascular lithotripsy has been used to modify coronary and peripheral calcium. Recent case reports have described the feasibility and safety of calcium modification in the aortic and mitral valves prior to device implantation. In our case, a high risk of aortic annular rupture was considered due to the high amount of calcium in a critical area involving the annulus and its transition to the LVOT. The use of both self-expandable and balloon-expandable valves has been described in heavily calcified aortic stenosis. However, balloon-expandable valves have advantages. In particular, they can facilitate better expansion and precise positioning during deployment. Two case reports have shown the use of IVL in the aortic annulus prior to TAVR, both with successful outcomes. In one report, a 29-mm Sapien S3 (Edward Lifesciences, Irvine, CA, USA) was deployed in the bicuspid aortic valve after IVL with three 7.0 mm × 60-mm balloons (Shockwave Medical, Santa Clara, CA, USA). In the other case, the IVL was used to perform an undersized valvuloplasty to advance an Amplatz left catheter into the left ventricle to place a stiff wire. The procedure was completed by conducting valvuloplasty with an 18 mm × 40-mm balloon and implanting a 26-mm Evolut-R valve (Medtronic, St. Paul, Minnesota, MN, USA), and by optimizing the final result by postdilating the valve with a conventional valvuloplasty balloon.^1–3^

To the best of our knowledge, this is the first case report involving the use of Accurate Neo-2 (Boston Scientific, Marlborough, MA, USA). In our case, a self-expandable device was selected for supra-annular positioning, since the patient had an extremely small annulus. In this context, we were aware of the high risk of under-expansion, which requires an aggressive predilatation to create space and prevent recoil. Therefore, this case required a self-expandable *trans*-catheter heart valve with a high radial force. Considering these features, Evolut Fx (Medtronic, St. Paul, Minnesota, MN, USA) was the primary choice. However, Accurate Neo-2 was the only self-expandable device available at our centre at that time.

**Figure 5:**
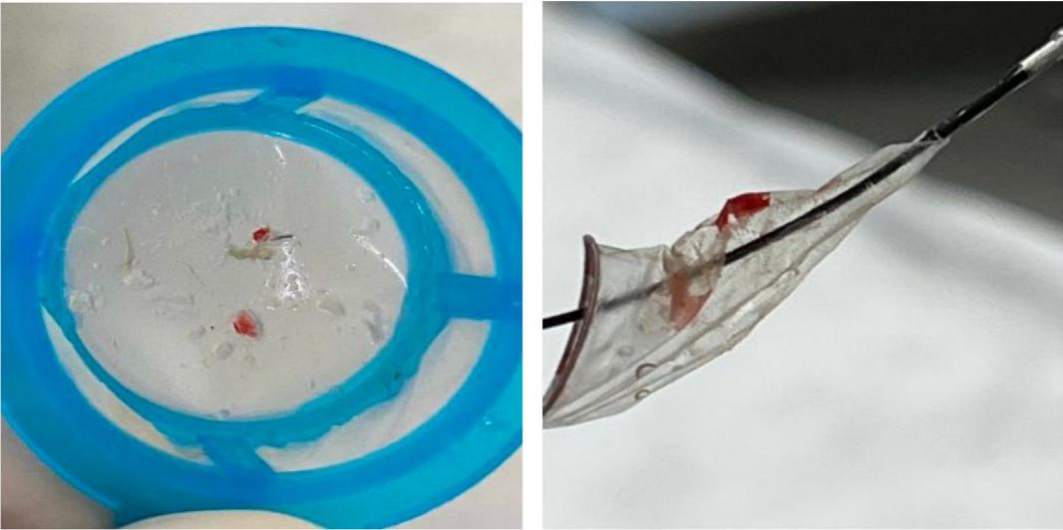
Sentinel device exposing the debris captured.

The use of a cerebral embolic protection device, with evidence of debris postprocedure, emphasized the extensive manipulation of the aortic annulus during IVL therapy and subsequent balloon dilatation (*Figure 5*). These protective devices reduce the risk of peri-procedural clinical ischaemic events and the burden posed by new silent ischaemic lesions during TAVR.^7^

There are currently no recommendations regarding the optimal diameter for aortic valve preparation with IVL balloons. Based on our knowledge about coronary angioplasty, we applied the diameter-selection principles used for hugging and kissing balloons, combined with Finet´s formula for the aortic annulus diameter, as a theoretical guide to achieve optimal contact with the existing calcium with a three-balloon strategy. Our patient had an annulus diameter of 15.8 mm × 20.7 mm (with a mean diameter of 18.2 mm) on CT scan. Consequently, we applied Finet’s formula (D1 = 0.678 × [D2 + D3]) to the selected balloon diameters, adapted for three balloons as follows: annulus diameter = 0.678 × (balloon 1 diameter + balloon 2 diameter + balloon 3 diameter)]. Using selected balloon sizes (7 mm + 7 mm + 8 mm), the calculated effective diameter was 14.91 mm, with a target annular mean diameter of approximately 18.2 mm, while maintaining a low risk of rupture and objectifying a decrease in the peak-to-peak gradient. Then, to confirm calcium modification and to achieve a better valve expansion, dilatation was performed with an 18 mm × 40-mm valvuloplasty balloon, which was adequately expanded under fluoroscopic guidance.

The current case emphasizes the novel use of calcium-modifying devices beyond coronary and peripheral vessels with promising outcomes. This approach could expand the viability to manage severe aortic stenosis with a large amount of calcium in the aortic annulus, potentially allowing percutaneous aortic valve replacement with a lower risk of annular rupture. Our case illustrates the feasibility of using a self-expandable valve in this setting, as effective calcium modification can overcome the lower radial force of these devices. Although the valve has now been discontinued, it was approved worldwide at the time of the procedure. Further research on IVL aortic valvuloplasty, including the development of a dedicated device for this type of adjunct therapy and the establishment of evidence-based recommendations for its clinical use, should be performed.

## Lead author biography


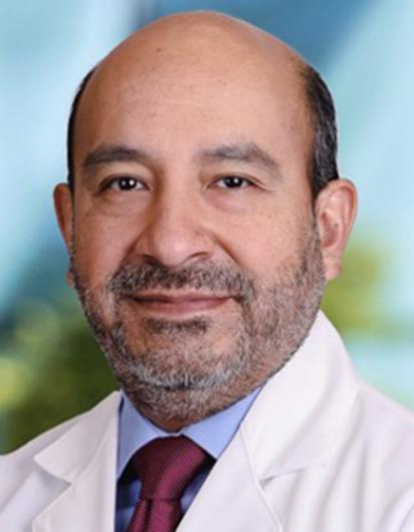


Graduated in cardiology and interventional cardiology from the “Ignacio Chávez” National Institute of Cardiology in Mexico. Master’s degree in science at the UNAM, a Master’s degree in structural interventions at the Francisco de Vitoria University in Madrid, Spain and a postdoctoral training in CHIP, peripheral vascular, and valvular interventions at the Cardiovascular Institute of Paris and the Cardiovascular Center of Eastern France. He is a professor of interventional cardiology at the Cardiology Hospital of the National Medical Center XXI Century, Mexico and is the current vice president of the Mexican Society of Interventional Cardiology.

## Data Availability

The data underlying this article will be shared upon reasonable request to the corresponding author.
